# Efficacy and safety of aerosolized intra-tracheal dornase alfa administration in patients with SARS-CoV-2-induced acute respiratory distress syndrome (ARDS): a structured summary of a study protocol for a randomised controlled trial

**DOI:** 10.1186/s13063-020-04488-8

**Published:** 2020-06-19

**Authors:** J. P. Desilles, C. Gregoire, C. Le Cossec, J. Lambert, O. Mophawe, M. R. Losser, F. Lambiotte, S. Le Tacon, M. Cantier, N. Engrand, P. Trouiller, J. Pottecher

**Affiliations:** 1 Biological Resource Center, Interventional Neuroradiology Department, Rothschild Foundation Hospital, Paris, France; 2 Laboratory of Vascular Translational Science, U1148 INSERM, Université de Paris, Paris, France; 3 Program in Cellular and Molecular Medicine, Boston Children’s Hospital, Harvard Medical School, Boston, MA 02115 USA; 4 Intensive Care Department, Rothschild Foundation Hospital, Paris, France; 5 Clinical Research Unit, Rothschild Foundation Hospital, Paris, France; 6 CHRU Nancy, Pôle d’Anesthésie-Réanimation, 29 Avenue de Lattre de Tassigny, 54000 Nancy, France; 7 Service de Réanimation Polyvalente, Centre Hospitalier de Valenciennes, Avenue Désandrouin, 59322 Valenciennes, France; 8 CHR Metz-Thionville-Site de Mercy, Service de Réanimation Polyvalente, 1 Allée du Château, 57350 Ars-Laquenexy, France; 9 Hôpitaux Universitaires de Strasbourg, Hôpital de Hautepierre, Service d’Anesthésie-Réanimation Chirurgicale, 1 Avenue Molière, 67098 Strasbourg, France; 10 Université de Strasbourg, Faculté de Médecine, Fédération de Médecine Translationnelle de Strasbourg (FMTS), EA3072, Strasbourg, France

**Keywords:** COVID-19, Randomised controlled trial, Lung damage, Neutrophil extracellular traps, ARDS, Dornase alfa

## Abstract

**Objectives:**

Severe Acute Respiratory Syndrome Coronavirus 2 (SARS-CoV-2) may trigger severe pneumonia in coronavirus disease of 2019 (COVID-19) patients through release of damage-associated molecular patterns (DAMPs) and recruitment of neutrophils in the lungs. Activated neutrophils induce inflammation and severe alveolar injury by releasing neutrophil extracellular traps (NETs). The backbones of many DAMPs and NETs are made of extracellular, cell-free DNA decorated with highly toxic compounds such as elastase, myeloperoxidase and citrullinated histones. Dornase alfa is a FDA-approved recombinant human DNAse 1 for the treatment of cystic fibrosis, which cleaves extracellular DNA and may break up cell-free DNA, loosening sticky mucus in the distal airways and reducing NETs-induced toxicity on alveolar pneumocytes. The COVIDornase trial intends to define the impact of aerosolized intra-tracheal dornase alfa administration on the severity and progression of acute respiratory distress syndrome (ARDS) in COVID-19 patients. This drug might make lung mucus thinner and looser, promoting improved clearance of secretions and reduce extracellular double-stranded DNA-induced hyperinflammation in alveoli, preventing further damage to the lungs.

**Trial design:**

COVIDornase is a prospective, randomized, controlled, 2-arm (1:1 ratio), multicentric, open-label clinical trial.

**Participants:**

The study will recruit mechanically ventilated patients hospitalized in the intensive care unit (ICU) in the recruiting centres (at the time of writing: The Rothschild foundation hospital in Paris, the Strasbourg university hospitals, and Metz-Thionville hospital) who have been diagnosed with COVID-19 and meet ARDS criteria.

**Inclusion criteria:**

- Adult patient (age ≥ 18 years old);

- Hospitalized in ICU;

- With severe COVID-19 pneumonia and ARDS according to Berlin criteria (PaO_2_/FiO_2_ < 300 and PEEP > 5 cmH_2_O);

- Intubated for less than 8 days;

- With an anticipated duration of mechanical ventilation > 48 hours;

- Carrier of an arterial catheter;

- For whom 4 PaO_2_/FiO_2_ values over the preceding 24 hours are available;

**Non-inclusion criteria:**

- Known hypersensitivity to dornase alfa or any of its excipients;

- Pregnant or breastfeeding status;

- Patient under legal protection.

**Intervention and comparator:**

**Intervention 1,** Study group

Dornase alfa (Pulmozyme®, Roche, Switzerland) will be administered by aerosol, at a dose of 2500 IU twice daily, 12 hours apart, for 7 consecutive days, using a vibrating mesh nebulizer (Aerogen Solo®, Aerogen, Ireland). The remainder of the management will be performed in accordance with good clinical practice, including mechanical ventilation (protective ventilation, PEEP > 5 cmH_2_O, tracheal balloon pressure check every 4 hours or automatic device, 30° head of the bed elevation, tidal volume 6-8mL/kg, plateau pressure < 30 cmH_2_O), neuromuscular blockers if necessary, prone position if PaO_2_/FiO_2_ < 150, early enteral nutrition, glycemic control and a sedation protocol based on the RASS score.

**Intervention 2,** Comparator

Patients will receive usual care in accordance with good practice (as detailed above), without aerosols.

**Main outcomes:**

The primary outcome is the occurrence of at least one grade improvement between D_0_ (inclusion) and D_7_ in the ARDS scale severity (Berlin criteria). For instance from “severe” to “moderate” or from “moderate” to “mild”.

**Randomisation:**

All consecutive patients meeting the inclusion criteria will be randomised 1:1 using an eCRF-based, computer-generated randomisation table, either to the dornase alfa arm or to the control arm. An interim analysis will be performed after inclusion of 20 patients. Inclusions may be stopped at the interim analysis per data safety and monitoring board (DSMB) advice, if statistical analyses conclude on the futility or efficacy of the intervention or by other DSMB decision.

**Blinding (masking):**

The participants and caregivers will not be blinded to study group assignment. Those assessing the outcomes will be blinded to study group assignment.

**Numbers to be randomised (sample size):**

Fifty patients will be randomized to each group, 100 patients in total.

**Trial Status:**

Protocol version number 2, April 29^th^, 2020. Recruitment is ongoing. The trial started recruitment on the 21^st^ April 2020. We estimate recruitment will finish August 21^st^ 2020.

**Trial registration:**

The trial was registered in ClinicalTrials.gov on 21 April 2020, updated on 8 May 2020. Trial registration number is NCT04355364.

**Full protocol:**

The full protocol is attached as an additional file, accessible from the Trials website (Additional file 1). In the interest in expediting dissemination of this material, the familiar formatting has been eliminated. This Letter serves as a summary of the key elements of the full protocol.

## Supplementary information

**Additional file 1.** Full Study Protocol.

## Data Availability

Study documents will be de-identified, stored in each recruitment centre and kept for at least 15 years in a locked, secure office, according to French law. All personnel involved in data analysis will be masked. Only the principal investigators, the DSMB and the statisticians will have access to the final data set. The data sets used and analysed during the current study will be available from the corresponding author on reasonable request, after publication of the main core article.

