# Supplementary material for: Efficacy and safety of aerosolized intra-tracheal dornase alfa administration in patients with SARS-CoV-2-induced acute respiratory distress syndrome (ARDS): a structured summary of a study protocol for a randomised controlled trial
Source: Trials. 2020 Jun 19;21:548. doi: 10.1186/s13063-020-04488-8 (PMC7303591; doi:10.1186/s13063-020-04488-8)
Supplement: Supplementary file 1 — Additional file 1. Full Study Protocol. [file 13063_2020_4488_MOESM1_ESM.pdf]

HÔPITAL FONDATION
Adolphe de ROTHSCHILD
LA RÉFÉRENCE TÊTE ET COU

PROTOCOLE DE RECHERCHE INTERVENTIONNELLE PORTANT SUR UN MÉDICAMENT
COVID-19 - COVIDornase<br>Intérêt de l'administration de Dornase alpha en aérosol dans le SDRA secondaire à une infection respiratoire par le coronavirus SARS-CoV-2 – COVID-19

Code de la recherche : CGE_2020_9

N° EUDRACT : 2020-001492-33

Version du protocole n° 2 du 29/04/2020

Avis favorable du CPP ouest IV Nantes le 08/04/2020

Autorisation de l'ANSM le 12/04/2020

**Promoteur**	**Fondation Adolphe de Rothschild**<br>29 rue Manin<br>75019 Paris<br>**Représentant du promoteur :**<br>Dr Amélie Yavchitz, Cheffe de Service Adjointe<br>Service de Recherche Clinique / DRCI<br>Tél : 01 48 03 64 54. Fax : 01 48 03 64 30<br>Mail : ayavchitz@for.paris

**Investigateur *coordonnateur/principal***	Dr Charles Grégoire<br>Service de Réanimation<br>Hôpital Fondation Adolphe de Rothschild<br>Tél : 01 48 03 69 85<br>Mail : cgregoire@for.paris

**Responsable scientifique**	Dr Jean-Philippe Désilles<br>Centre de Ressources Biologiques<br>Service de Neuroradiologie interventionnelle<br>Hôpital Fondation Adolphe de Rothschild<br>Tel : 06 72 87 29 06<br>Mail : jpdesilles@for.paris

**Méthodologiste**	Chloé Le Cossec<br>Service de Recherche clinique / DRCI<br>29 rue Manin, 75019 Paris<br>Mail : clecossec@for.paris

**Responsable pharmacovigilance**	Sophie Houdas<br>Service de Recherche Clinique / DRCI<br>29 rue Manin, 75019, Paris<br>Mail : shoudas@for.paris

Ce document confidentiel est la propriété de la Fondation Adolphe de Rothschild. Aucune information non publiée figurant dans ce document ne peut être divulguée sans autorisation écrite préalable de la Fondation Adolphe de Rothschild.

PAGE DE SIGNATURE DU PROTOCOLE

Titre et acronyme de l'étude :
COVIDornase : Intérêt de l'administration de Dornase alpha en aérosol dans le SDRA secondaire à une infection respiratoire par le coronavirus SARSCoV-2

Code de la recherche : CGE_2020_9

Version du protocole n° 2 du 29/04/2020

Ce protocole a été lu et approuvé à la date notée ci-dessous.

Les deux parties s'engagent à mener la recherche conformément au protocole, aux bonnes pratiques cliniques, et aux dispositions législatives et réglementaires en vigueur.

POUR LE PROMOTEUR :

Dr Amélie Yavchitz, Cheffe de Service Adjointe
Service de Recherche Clinique / DRCI
Fondation Adolphe de Rothschild

Date : 29/04/2020
Signature :

L'INVESTIGATEUR *COORDONNATEUR/PRINCIPAL* :

Dr Charles Grégoire
Service de Réanimation
Fondation Adolphe de Rothschild

Date : 29/04/2020
Signature :

TABLE DES MATIERES

1 Introduction - justification scientifique 11
2 Objectifs de la recherche 14
2.1 Hypothèse testée 14
2.2 Objectif principal 14
2.3 Objectifs secondaires 14
3 Critères de jugement 15
3.1 Critère de jugement principal 15
3.2 Critères de jugement secondaires 15
4 Sélection et non inclusion des personnes de la recherche 16
4.1 Population cible et mode de recrutement 16
4.2 Critères d'inclusion 16
4.3 Critères de non inclusion 16
5 Description de l'étude 17
5.1 Type d'étude 17
5.2 Déroulement de la recherche 17
5.2.1 Centres investigateurs 17
5.2.2 Modalités de recrutement 17
5.2.3 Information des patients et recueil de leur consentement 17
5.2.4 Modalités de prise en charge des patients 17
5.2.5 Modalités de sorties d'étude 19
5.2.6 Recueil des données 20
5.2.7 Tableau récapitulatif du déroulement de l'étude 20
5.3 Calendrier de l'étude 21
5.4 Description des mesures prises pour réduire et éviter les biais 22
5.5 Bénéfices, risques et contraintes pour le patient participant à la recherche 22
6 Description des médicaments de l'étude 24
6.1 Identification du traitement 24
6.2 Conditionnement et étiquetage 24
6.3 Fabrication et distribution des traitements 24
6.4 Administration 25
7 Evaluation de la sécurité 26
7.1 Définitions 26
7.2 Enregistrement et notification des évènements indésirables par l'investigateur 28
7.2.1 Evènements indésirables 28
7.2.2 Evènements indésirables graves 28
7.2.3 Evènements indésirables d'intérêt particulier 29
7.3 Prise en charge des évènements indésirables graves par le promoteur 29
7.3.1 Evaluation médicale 29
7.3.2 Déclaration des suspicions d'effets indésirables graves inattendus (SUSAR) 29
7.4 Fait nouveau et mesures urgentes de sécurité 30
7.5 Rapport annuel de sécurité 30
7.6 Comité de surveillance indépendant 30
8 Analyses statistiques 31
8.1 Calcul du nombre de sujets 31
8.2 Description des analyses statistiques utilisées 31
8.2.1 Choix des personnes à inclure dans les analyses 31
8.2.2 Statistiques descriptives 31
8.2.3 Comparaisons des groupes à la baseline 31
8.2.4 Analyse du critère de jugement principal 31
8.2.5 Analyse des critères de jugement secondaires 32
8.3 Niveau de significativité statistique 34

8.4 Modalités de prise en compte des données manquantes, non utilisées ou non valides............34
8.5 Gestion des modifications apportées au plan statistique initial..34
9 Dispositions réglementaires..35
9.1 Demande d'autorisation auprès de l'autorité compétente (Agence Nationale de Sécurité du Médicament et des produits de santé - ANSM)..35
9.2 Demande d'avis auprès du Comité de Protection des Personnes (CPP)..................................35
9.3 Engagements – aspects éthiques..35
9.4 Déclaration du démarrage et de la fin de l'étude...36
9.5 Note d'information et recueil du consentement écrit du patient...36
9.6 Modifications du protocole..36
9.7 Déclaration CNIL (Commission Nationale de l'Informatique et des Libertés)........................37
9.8 Droits d'accès aux données et documents sources...37
9.9 Archivage..38
10 Assurance et financement..39
10.1 Assurance..39
10.2 Financement..39
11 Règles relatives à la publication et rapport final..39
11.1 Enregistrement de l'étude...39
11.2 Rapport final..39
11.3 Communication et publication des résultats..40
12 Références..41

LISTE DES ABRÉVIATIONS

ANSM : Agence Nationale de Sécurité du Médicament et des produits de santé

CNIL : Commission Nationale de l'Informatique et des Libertés

CPP : Comité de Protection des Personnes

CSI : Comité de Surveillance Indépendant

e-CRF : Electronic Case Report Form

EI : Evénement Indésirable

EIG : Événement Indésirable Grave

RGPD : Règlement Général sur la Protection des Données de l'Union européenne

SUSAR : Suspected Unexpected Serious Adverse Reaction

SARSCoV-2 : Severe Acute Respiratory Syndrome-Coronavirus 2

OMS: organisation mondiale de la santé

COVID-19 : Coronavirus Disease 2019

SDRA : Syndrome de Détresse Respiratoire Aigue

DNAse : Désoxiribonucléase

NETs : Neutrophil Extracellular Traps

AMM : Autorisation de Mise sur le Marché

PNN : Polynucléaire Neutrophile

VM : ventilation mécanique

SYNOPSIS

**Titre de la recherche**	Intérêt de l'administration de Dornase alpha en aérosol dans le SDRA secondaire à une infection respiratoire par le coronavirus SARS-CoV-2 – COVID-19
**Acronyme**	**COVID-19 – COVIDornase**
**Promoteur**	Hôpital Fondation Adolphe de Rothschild
**Investigateur principal**	Dr Charles Grégoire
**Nombre de centres**	2 à 5 (intervalle dans lequel d'autres centres pourront être ouverts)
**Justification de la recherche**	Le syndrome de détresse respiratoire aigüe (SDRA) est la forme la plus grave des infections COVID-19 avec près de 70% de mortalité. A ce jour, aucune thérapeutique spécifique n'a montré son efficacité. Lors d'une infection respiratoire virale, le poumon est le siège d'un important recrutement de polynucléaires neutrophiles (PNN). Les PNN recrutés génèrent la formation de NETs (neutrophil extracellular traps) au sein des alvéoles et des bronchioles. Il a été mis en évidence que les NETs participaient à l'encombrement broncho-alvéolaire et à l'amplification de la réponse inflammatoire au cours des pneumonies virales responsable du SDRA. La DNAse 1 est une enzyme capable de couper les brins d'ADN extracellulaire, véritable colonne vertébrale des NETs. L'administration de DNAse 1 (dornase alpha) entraine la fluidification du mucus broncho-alvéolaire ainsi qu'une réduction de la réponse inflammatoire au sein des alvéoles.<br>Nous souhaitons mener un essai randomisé contrôlé, bicentrique, en ouvert, afin d'évaluer l'efficacité de la dornase alpha en administration intra-trachéale chez les patients sous ventilation mécanique, hospitalisés en réanimation pour SDRA lié au COVID-19.
**Objectif et critère de jugement principal**	**<u>Objectif principal :</u>**<br>Evaluer l'efficacité de l'administration intratrachéale de dornase alfa (Pulmozyme©) sur l'évolution des paramètres ventilatoires à J7.<br>**<u>Critère principal :</u>**<br>Comparaison entre les deux bras de traitement du pourcentage de patients qui se sont améliorés d'au moins un grade sur l'échelle des SDRA (critères de Berlin) entre J0 (inclusion) et J7.
**Objectifs et critères de jugement secondaires**	**Objectifs secondaires :**<br>Les objectifs secondaires sont de comparer entre les deux bras de randomisation :<br>1) l'amélioration du rapport PaO2/FiO2 entre J0 et J7<br>2) la mortalité toute cause à J28<br>3) l'évolution clinique à J28 ;<br>4) le délai jusqu'à amélioration de l'évolution clinique telle que définie pour le second critère de jugement secondaire.<br>5) le délai jusqu'à amélioration du SDRA telle que définie par le critère de jugement principal;<br>6) le nombre de jours vivant sans ventilation mécanique entre J0 et J28<br>7) le nombre de jours vivant hors réanimation entre J0 et J28 ;<br>~~8)~~les concentrations des NETs et des marqueurs sanguins de l'inflammation à différents temps<br>~~9)~~ les concentrations des NETs et des marqueurs de l'inflammation au sein des sécrétions bronchiques à différents temps

	10) la qualité de vie des patients survivants à J28<br>11) la survenue d'événements indésirables ;<br>**<u>Critères de jugement secondaires :</u>**<br>1) pourcentage de patients avec une amélioration du rapport PaO2/FiO2 d'au moins 50 entre J0 et J7<br>2) la mortalité toute cause à J28<br>3) Amélioration d'au moins deux points sur une échelle ordinale à 7 niveaux (d'après Cao et al. 2020), entre J0 et J28 (ou sortie d'hospitalisation);<br>4) le délai jusqu'à évolution clinique favorable telle que définie pour le critère précédent.<br>5) Délai jusqu'à amélioration du SDRA telle que définie pour le critère de jugement principal (jours) ;<br>6) Nombre de jours vivant sans ventilation mécanique ~~à~~entre J0 et J28<br>7) Nombre de jours vivant hors de réanimation entre J0 et J28 ~~(jours)~~ ;<br>8) Concentrations des NETs et des marqueurs sanguins de l'inflammation à différents temps (J0, J2, J7 et J28 ou sortie d'hospitalisation) : Leucocytes, neutrophiles, lymphocytes, plaquettes, Ferritine, Fibrinogène, CRP, PCT, LDH, D-dimères, NETs (ELISA H3cit, ADN libre, ELISA sandwich ADN-MPO), activité élastase neutrophilaire, Concentration MPO, MMP-9, activité DNAse, complexes TAT, PAI-1, Platelet factor 4, active and total TGF-β1, protéine basique du surfactant de type D, KL-6 (Kerbs Von Lungren 6), sRAGE, ADN mitochondrial, HSP70, HMGB-1<br>9) Concentrations des NETs et des marqueurs de l'inflammation au sein des sécrétions bronchiques à différents temps (J0, J2, J7 et J28 sortie d'hospitalisation) : RT-PCR SARS-CoV-2, NETs (ELISA H3cit, ADN libre, ELISA sandwich ADN/MPO), Activité élastase neutrophilaire, Concentration MPO, MMP-9, Activité DNAse, Active and total TGF-β1<br>10) Comparaison entre les deux bras de traitement du score moyen de l'échelle QoR-15F des patients survivants à J28<br>11) Taux d'événements indésirables et d'événements indésirables graves.
**Plan expérimental**	Essai randomisé, contrôlé, multicentrique, en ouvert.
**Population concernée**	Patients sous ventilation mécanique, hospitalisés en réanimation pour un SDRA, secondaire à une infection par COVID-19.
**Critères d'éligibilité**	**<u>Critères d'inclusion :</u>**<br>- Patient majeur (âge ≥ 18 ans) ;<br>- Hospitalisé en réanimation ;<br>- Atteint d'une pneumonie grave COVID-19 (diagnostic positif par RT-PCR sur prélèvement nasopharyngé ou respiratoire profond et/ou scanner thoracique évocateur : opacités en verre dépoli, consolidation, réticulation, épaississement des septa interlobulaires, nodules périphériques) avec critères de SDRA selon les critères de Berlin (PaO2/FiO2<300 et PEP>5).<br>- Intubé depuis moins de 8 jours ;<br>- Dont la durée prévisible de ventilation mécanique est > 48h ;<br>- Porteur d'un cathéter artériel ;<br>- Pour lequel 4 valeurs de PAO2/FiO2 sur sang artériel sur les 24 dernières heures sont disponibles ;<br>- Affilié ou bénéficiaire d'un régime de protection sociale d'assurance maladie ;<br>**<u>Critères de non-inclusion :</u>**

	- Hypersensibilité connue à la Dornase alfa ou à l'un des excipients ;<br>- Femme enceinte ou allaitant ;<br>- Patient bénéficiant d'une mesure de protection juridique.
**Traitements à l'essai**	**<u>Groupe expérimental :</u>** Dornase alfa recombinante (Pulmozyme®, Roche, 2500UI/2,5mL solution pour inhalation par nébulisation).<br>La dornase alfa sera administrée en inhalation, à la dose de 2500UI deux fois par jour, à 12 heures d'intervalle, pendant 7 jours consécutifs.<br>**<u>Groupe contrôle :</u>** prise en charge habituelle selon les recommandations
**Déroulement pratique de l'essai**	Le recrutement des patients sera réalisé dans les services de réanimation dédiés à la prise en charge des patients atteints d'une pneumonie grave COVID-19 des centres participant.<br><br>Compte-tenu, d'une part de l'état clinique du patient (forte probabilité qu'il soit en incapacité d'exprimer son consentement) et d'autre part de l'urgence de sa prise en charge, une procédure d'inclusion en urgence sera mise en place. Les visites des proches à l'hôpital n'étant actuellement pas autorisées pour les patients, la recherche d'un consentement écrit ne sera pas envisageable. Une information par téléphone d'un proche ou de la personne de confiance sera réalisée autant que possible, avec la recherche d'un consentement oral. Lorsque le patient aura repris un état de conscience suffisant, son consentement à la poursuite de l'étude et au traitement des informations recueillies sera recherché.<br><br>Les patients vérifiant les critères d'inclusion et de non-inclusion inclus dans l'étude seront randomisés (1:1) dans le bras contrôle (prise en charge selon les recommandations habituelles) ou dans le bras expérimental (dornase alfa). La randomisation sera stratifiée sur le centre et sur le rapport PAO2/FiO2 à l'inclusion (200 < PaO2/FiO2 ≤ 300 ; 100 < PaO2/FiO2 ≤ 200 ; PaO2/FiO2 ≤ 100).<br><br>Dans le groupe expérimental, le traitement à l'essai sera administré en inhalation, à la dose de 2500UI deux fois par jour, à 12 heures d'intervalle, pendant 7 jours consécutifs, à l'aide d'un nébuliseur à tamis vibrant. Le reste de la prise en charge sera réalisée conformément aux bonnes pratiques, notamment pour ce qui concerne la ventilation mécanique (ventilation protectrice, PEP > 5 cmH2O, vérification de la pression du ballonnet trachéal toutes les 4h ou dispositif automatique, position proclive à 30°, volume courant 6-8mL/kg, Pplat<30cmH2O), curarisation si nécessaire, séances de décubitus ventral si PaO2/FiO2<150, nutrition entérale précoce, contrôle glycémique, un protocole de sédation fondé sur le score RASS (objectif -5).<br><br>Dans le groupe contrôle, les patients bénéficieront de la prise en charge habituelle, conformément aux bonnes pratiques.<br><br>Les patients seront suivis jusqu'à J28 après la randomisation (ou jusqu'à leur sortie de l'hôpital ou leur décès le cas échéant).
**Nombre de patients**	Il s'agit d'une étude pilote pour laquelle nous ne disposons pas des hypothèses nécessaires à la réalisation d'un calcul de nombre de sujets à inclure. En tenant compte du recrutement potentiel des centres, nous estimons le potentiel d'inclusion à 100 patients maximum soit 50 patients/bras.

**Méthode statistique**	La comparaison des entre les groupes sera réalisée à l'aide d'une régression logistique ajustée sur les critères de stratification et sur l'administration concomitante de traitements anti-infectieux et anti-inflammatoires.
**Calendrier prévisionnel**	Durée de la période d'inclusion : 3 mois<br>Durée de participation de chaque sujet : 28 jours<br>Durée totale de l'étude : 4 mois
**Références**	1. Guan W, Ni Z, Hu Y, Liang W, Ou C, He J, et al. Clinical Characteristics of Coronavirus Disease 2019 in China. N. Engl. J. Med. 2020;NEJMoa2002032.<br>2. Acute Respiratory Distress Syndrome: The Berlin Definition. JAMA. 2012 doi=10.1001/jama.2012.5669<br>3. Narasaraju T, Yang E, Samy RP, Ng HH, Poh WP, Liew A-A, et al. Excessive Neutrophils and Neutrophil Extracellular Traps Contribute to Acute Lung Injury of Influenza Pneumonitis. Am. J. Pathol. 2011;179:199–210.<br>4. Porto BN, Stein RT. Neutrophil Extracellular Traps in Pulmonary Diseases: Too Much of a Good Thing? Front. Immunol. 2016<br>5. Zitter JN, Maldjian P, Brimacombe M, Fennelly KP. Inhaled Dornase alfa (Pulmozyme) as a noninvasive treatment of atelectasis in mechanically ventilated patients. J. Crit. Care. 2013;28:218.e1-218.e7.

COVIDornase

1 Introduction - justification scientifique

Fin 2019, un nouveau coronavirus, responsable d'un syndrome de détresse respiratoire aigu sévère (Severe Acute Respiratory Syndrome-Coronavirus 2, SARSCoV-2), a provoqué une épidémie de maladie respiratoire aiguë à Wuhan, en Chine.[1] L'Organisation mondiale de la santé (OMS) a appelé cette maladie « Coronavirus Disease 2019 » (COVID-19).

Le 30 Mars 2020, cette épidémie est devenue une pandémie affectant officiellement plus de 757 000 personnes dans le monde et responsable de plus de 37 000 morts dont près de 3000 en France.

Les signes cliniques initiaux les plus fréquents du COVID-19 sont ceux d'une infection respiratoire aigüe, avec fièvre et toux. L'évolution peut être marquée par l'apparition d'une dyspnée, pouvant évoluer vers un syndrome de détresse respiratoire aigüe (SDRA) dans environ 15% des cas. Le SDRA est un processus inflammatoire pulmonaire induisant un œdème pulmonaire non hydrostatique. Il est défini par la présence dans les 7 jours suivant une pathologie pulmonaire ou extra-pulmonaire aigüe par l'association d'une hypoxémie aigüe ($PaO_2/FiO_2 \leq 300$ mmHg) chez un patient ventilé avec une pression expiratoire positive (PEP) de 5 cmH_2O au moins, ainsi que d'infiltrats radiologiques bilatéraux non entièrement expliqués par une insuffisance cardiaque ou une surcharge volémique. La définition de Berlin distingue les SDRA selon le rapport PaO_2/FiO_2 en SDRA légers ($200 < PaO_2/FiO_2 \leq 300$ mmHg), SDRA modérés ($100 < PaO_2/FiO_2 \leq 200$ mmHg) et SDRA sévères ($PaO_2/FiO_2 \leq 100$ mmHg).[2]

A ce jour, aucune thérapeutique spécifique n'a montré son efficacité dans le COVID-19. La prise en charge des infections respiratoires graves liés au COVID-19 est donc principalement basée sur des soins de support et symptomatiques comprenant notamment la nécessité d'un ventilation mécanique (VM) prolongée. Le taux de mortalité du SDRA lié au COVID-19 est très important de l'ordre de 70%.

Les polynucléaires neutrophiles (PNN) sont la première ligne de défense lors d'une infection virale. Par leurs effets phagocytaires et leur activité antivirale, ils sont chargés de protéger et de maintenir l'intégrité de l'organisme face à une infection. Une fois activés, les PNN peuvent libérer des structures de chromatine appelées « neutrophil extracellular traps » (NETs) décorées d'histones citrullinés et de protéines granulaires cytoplasmiques (élastase neutrophilaire et myélopéroxydase notamment).[3] La formation des NETs a été initialement décrite comme un mécanisme de défense de l'hôte pour capturer et tuer les bactéries et les virus. Il est désormais bien établi que la formation des NETs a des conséquences beaucoup plus complexes. Notamment, la formation excessive de NETs ou une diminution de leur élimination peut favoriser la persistance d'une inflammation voire une destruction tissulaire après résolution de l'infection. Ces dernières années, les NETs ont été reconnus comme étant impliqués dans la physiopathologie de plusieurs maladies pulmonaires, notamment la

mucoviscidose ou les pneumopathies infectieuses et l'évolution vers une fibrose. L'aspect caractéristique d'une inflammation du poumon suivant l'infection par la grippe est représentée par une abondante infiltration neutrophilaire au sein des alvéoles pulmonaires.[4] Dans une étude expérimentale de grippe H1N1, il a été montré qu'au sein du poumon la production de NETs satellite de l'infiltration des neutrophiles était délétère. Les NETs étaient retrouvés au sein des alvéoles, responsables d'une destruction des capillaires alvéolaires, mais également au sein des bronchioles avec des lésions et des obstructions des bronchioles (Figure).[5]

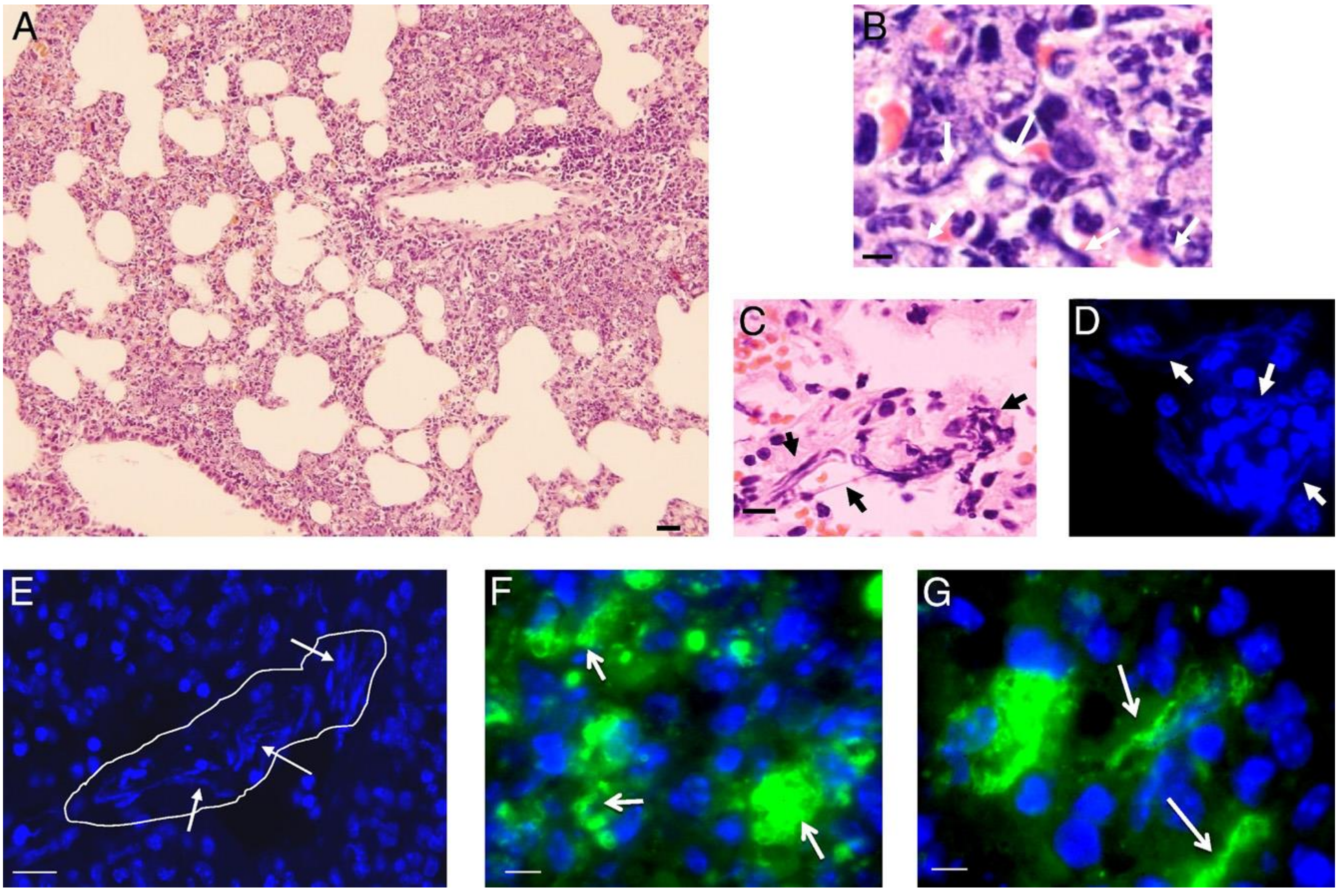

Figure : Mise en évidence in vivo dans un modèle de pneumonie H1N1 chez la souris de la formation de NETs au sein des alvéoles et des bronchioles. (Narasaraju T, et al. **Excessive Neutrophils and Neutrophil Extracellular Traps Contribute to Acute Lung Injury of Influenza Pneumonitis.** Am. J. Pathol. 2011)

Les effets délétères d'une accumulation de NETs a également été décrits au cours d'un SDRA, avec mise en évidence de NETs au sein des alvéoles pulmonaires provoquant des lésions pulmonaires étendues[6] et une hypoxémie. De plus, alors que l'élastase neutrophilaire (EN) libre est généralement rapidement inactivée lorsqu'elle est dans le plasma, l'EN liée à l'ADN au sein des NETs est protégée de la neutralisation.[7] En conséquence, son effet protéolytique persiste, responsable d'une réponse inflammatoire exacerbée et délétère pour le tissu pulmonaire.

Les fibres extracellulaires d'ADN double brin constituent la structure des NETs. L'ADN extracellulaire est physiologiquement décomposé par les désoxyribonucléases endogènes (DNAses) dont principalement la DNAse 1. Une DNAse 1 recombinante humaine est disponible dans le commerce depuis 1994 (dornase alfa, Pulmozyme; Roche, Bâle, Suisse et Genentech, San Francisco,

CA, USA) et dispose d'une AMM pour la traitement des exacerbations pulmonaires des patients porteurs d'une mucoviscidose. Des études *in vitro* ont suggéré que le traitement par dornase alfa entrainait une libération des protéases neutrophilaires,[8] ainsi qu'une solubilisation du mucus broncho-alvéolaire.[9]

La dornase alfa est administrée par voie intratrachéale (aérosols), ses actions biologiques et les propriétés pharmacocinétiques comportant notamment une excellente sécurité avec aucune complication grave rapportée[10] pourrait avoir un bénéfice pour l'amélioration clinique chez les patients présentant un SDRA secondaire au COVID-19. En effet, la dornase alfa s'est déjà avérée réduire les lésions pulmonaires dans les SDRA induits par une septicémie.[11] Enfin, dans un petit essai clinique randomisé, la dornase alfa en aérosol entrainait une amélioration de l'oxygénation chez les patients en soins intensifs en ventilation mécanique avec atélectasie lobaire.[12] Une étude très récente suggère enfin que les deux principaux facteurs de risque de développer un SDRA en cas d'infection par le SARSCoV-2 sont l'âge et le taux de neutrophiles sanguin à l'admission (Wu et al., JAMA. March 2020, in press), également connus pour favoriser la formation des NETs en cas d'infection aigue.

Résumé de l'intérêt et de l'originalité du projet

- Lors d'une infection respiratoire virale, le poumon est le siège d'un important recrutement de polynucléaires neutrophiles (PNN). Les PNN recrutés puis activés génèrent la formation de NETs au sein des alvéoles et des bronchioles.
- Il a été mis en évidence que les NETs participaient à l'encombrement broncho-alvéolaire et à l'amplification de la réponse inflammatoire au cours des pneumonies virales responsable du syndrome de détresse respiratoire aigu (SDRA).
- La DNAse 1 est une enzyme capable de couper les brins d'ADN extracellulaire, véritable colonne vertébrale des NETs
- L'administration de DNAse 1 (Dornase Alpha) entraine la fluidification du mucus broncho-alvéolaire ainsi qu'une réduction de la réponse inflammatoire au sein des alvéoles.
- L'hypothèse de l'étude est que l'administration intra-trachéale de Dornase Alpha chez les patients en SDRA lié au COVID-19 en ventilation mécanique permettrait d'améliorer les paramètres ventilatoires et ainsi de réduire la durée de ventilation mécanique.

2 Objectifs de la recherche

2.1 Hypothèse testée

L'administration par aérosols de Dornase alpha améliore les paramètres ventilatoires des patients en SDRA suite au COVID-19.

2.2 Objectif principal

Évaluer l'efficacité de l'administration intratrachéale de dornase alfa (Pulmozyme©) sur l'évolution des paramètres ventilatoires à J7.

2.3 Objectifs secondaires

Les objectifs secondaires sont de comparer entre les deux bras de randomisation :

1) l'amélioration du rapport PaO2/FiO2 entre J0 et J7
2)la mortalité toute cause à J28

3) l'évolution clinique à J28 ;

4) le délai jusqu'à amélioration de l'évolution clinique telle que définie pour le second critère de jugement secondaire.

5) le délai jusqu'à amélioration du SDRA telle que définie par le critère de jugement principal

6) le nombre de jours vivant sans ventilation mécanique entre J0 et J28

7)-le nombre de jours vivant hors réanimation entre J0 et J28 ;

8) les concentrations des NETs et des marqueurs sanguins de l'inflammation à différents temps

9) les concentrations des NETs et des marqueurs de l'inflammation au sein des sécrétions bronchiques à différents temps

10) la qualité de vie des patients survivants à J28

11) la survenue d'événements indésirables ;

3 Critères de jugement

3.1 Critère de jugement principal

Comparaison entre les deux bras de traitement du taux de patients présentant une amélioration d'au moins un grade sur l'échelle de SDRA (moyenne des 4 valeurs de PaO2/FiO2 des 24 heures) entre J0 (inclusion) et J7 chez les patients atteints d'un SDRA secondaire à une infection COVID-19 hospitalisés en réanimation.

3.2 Critères de jugement secondaires

1) pourcentage de patients avec une amélioration du rapport PaO2/FiO2 d'au moins 50 entre J0 et J7

2)la mortalité toute cause à J28

3) Amélioration d'au moins deux points sur une échelle ordinale à 7 niveaux (d'après Cao et al. 2020), entre J0 et J28 (ou sortie d'hospitalisation);

4) le délai jusqu'à évolution clinique favorable telle que définie pour le critère précédent.

5) le délai jusqu'à amélioration du SDRA telle que définie pour le critère de jugement principal (jours) ;

6) Nombre de jours vivant sans ventilation mécanique entre J0 et J28

7) Nombre de jours vivant hors de réanimation entre J0 et J28;

8) Concentrations des NETs et des marqueurs sanguins de l'inflammation à différents temps (J0, J2, J7 et J28 ou sortie d'hospitalisation) : Leucocytes, neutrophiles, lymphocytes, plaquettes, Ferritine, Fibrinogène, CRP, PCT, LDH, D-dimères, NETs (ELISA H3cit, ADN libre, ELISA sandwich ADN-MPO), activité élastase neutrophilaire, Concentration MPO, MMP-9, activité DNAse, complexes TAT, PAI-1, Platelet factor 4, active and total TGF-β1, protéine basique du surfactant de type D, KL-6 (Kerbs Von Lungren 6), sRAGE, ADN mitochondrial, HSP70, HMGB-1

9) Concentrations des NETs et des marqueurs de l'inflammation au sein des sécrétions bronchiques à différents temps (J0, J2, J7 et J28 sortie d'hospitalisation) : RT-PCR SARS-CoV-2, NETs (ELISA H3cit, ADN libre, ELISA sandwich ADN/MPO), Activité élastase neutrophilaire, Concentration MPO, MMP-9, Activité DNAse, Active and total TGF-β1

10) Comparaison entre les deux bras de traitement du score moyen de l'échelle QoR-15F des patients survivants à J28

11) Taux d'événements indésirables et d'événements indésirables graves.

4 Sélection et non inclusion des personnes de la recherche

4.1 Population cible et mode de recrutement

Le recrutement des patients sera réalisé dans les services de réanimation de l'hôpital Fondation Adolphe de Rothschild et du CHU de Strasbourg dédiés à la prise en charge des patients atteints d'une pneumonie grave COVID-19.

4.2 Critères d'inclusion

- Patient majeur (âge ≥ 18 ans) ;
- Hospitalisé en réanimation ;
- Atteint d'une infection respiratoire grave COVID-19 (diagnostic positif par RT-PCR sur prélèvement nasopharyngé ou respiratoire profond et/ou scanner thoracique évocateur : opacités en verre dépoli, consolidation, réticulation, épaississement des septal interlobulaires, nodules périphériqueset/ou scanner thoracique) avec critères de SDRA selon les critères de Berlin (PaO2/FiO2<300 et PEP>5).
- Intubé depuis moins de 8 jours ;
- Dont la durée prévisible de ventilation mécanique est > 48h ;
- Porteur d'un cathéter artériel ;
- Pour lequel 4 valeurs de PAO2/FiO2 sur sang artériel sur les 24 dernières heures sont disponibles ;
- Affilié ou bénéficiaire d'un régime de protection sociale d'assurance maladie ;

4.3 Critères de non inclusion

- Hypersensibilité connue à la Dornase alfa ou à l'un des excipients ;
- Femme enceinte ou allaitant ;
- Patient bénéficiant d'une mesure de protection juridique.

5 Description de l’étude

5.1 Type d’étude

Essai randomisé, contrôlé, multicentrique, en ouvert.

5.2 Déroulement de la recherche

5.2.1 *Centres investigateurs*

- Hôpital Fondation Adolphe de Rothschild

- CHU de Strasbourg

D’autres centres pourront être ouverts en cours d’étude.

5.2.2 *Modalités de recrutement*

Le recrutement des patients sera réalisé dans les services de réanimation dédiés à la prise en charge des patients atteints d’une pneumonie grave COVID-19 des centres participant.

5.2.3 *Information des patients et recueil de leur consentement*

Compte-tenu, d’une part de l’état clinique du patient (forte probabilité qu’il soit en incapacité d’exprimer son consentement) et d’autre part de l’urgence de sa prise en charge, une procédure d’inclusion en urgence sera mise en place. Les visites des proches à l’hôpital n’étant actuellement pas autorisées pour les patients, la recherche d’un consentement écrit ne sera pas envisageable. Une information par téléphone d’un proche ou de la personne de confiance sera réalisée autant que possible, avec la recherche d’un consentement oral. Un délai de 12 heures sera proposé au proche pour donner son consentement ou non à l’étude proposée. Lorsque le patient aura repris un état de conscience suffisant, son consentement à la poursuite de l’étude et au traitement des informations recueillies sera recherché.
Si ce dernier n’a pas retrouvé un état de conscience suffisant, le consentement d’un proche (membres de la famille ou personne de confiance) à la poursuite de la recherche sera recherché dès que possible.

5.2.4 *Modalités de prise en charge des patients*

Les patients vérifiant les critères d’inclusion et de non-inclusion inclus dans l’étude seront randomisés (1:1) dans le bras contrôle (prise en charge selon les recommandations habituelles) ou dans le bras expérimental (dornase alfa).

Dans le groupe expérimental, le traitement à l'essai sera administré en inhalation, à la dose de 2500UI deux fois par jour, à 12 heures d'intervalle, pendant 7 jours consécutifs, à l'aide d'un nébuliseur à tamis vibrant. Une aspiration bronchique protégée sera systématiquement réalisée immédiatement avant chaque nébulisation. La nébulisation sera réalisée sur une durée courte (<10 minutes), en présence d'un infirmier dans la chambre du patient. Une aspiration bronchique protégée sera réalisée après la fin de la nébulisation en cas d'augmentation >20% des résistances mesurées par le respirateur sur les 10 minutes de surveillance post nébulisation. Le reste de la prise en charge sera réalisée conformément aux bonnes pratiques, notamment pour ce qui concerne la ventilation mécanique (ventilation protectrice, PEP > 5 cmH2O, vérification de la pression du ballonnet trachéal toutes les 4 heures ou dispositif automatique, position proclive à 30°, volume courant 6-8mL/kg, Pplat<30 cmH2O), curarisation si nécessaire, séances de décubitus ventral si PaO2/FiO2<150, la nutrition entérale précoce, le contrôle glycémique, un protocole de sédation fondé sur le score RASS (objectif -5).

Dans le groupe contrôle, les patients bénéficieront de la prise en charge habituelle, conformément aux bonnes pratiques.

Conformément à la prise en charge habituelle en réanimation, un cathéter artériel permettra la réalisation de gaz du sang artériels toutes les 6 heures.

Les patients seront suivis jusqu'à J28 après la randomisation (ou jusqu'à leur sortie de l'hôpital ou leur décès le cas échéant). Pour les patients sortis de l'hôpital, leur statut vital et ventilatoire sera recherché à J28.

Les traitements à visée anti-infectieuse ou anti-inflammatoire administrés de façon concomitante au traitement expérimental (de J0 à J7) ainsi que lors de la période de surveillance (J7 à J28) seront systématiquement colligés dans les cahiers d'observation.

Des prélèvements biologiques sanguins (4mL de sang sur tube EDTA et 3ml de sang citrate) et sécrétions bronchiques (pot stérile protégé) seront réalisés à J0, J2, J7 et J28 (ou à la sortie d'hospitalisation).

<u>Création d'une collection d'échantillons biologiques (bio-banque) : Les échantillon biologiques (sanguins et sécrétions bronchiques) collectés pour la réponse aux critères de jugement secondaires seront ensuite conservés pour</u> constitution d'une collection d'échantillons biologiques de patients COVID-19 en SDRA.

Ces prélèvements seront conservés à -80°C pour une durée maximale de 15 ans au Centre de Ressources Biologiques (CRB) de l'hôpital Fondation Adolphe de Rothschild ou au CRB des Hôpitaux Universitaires de Strasbourg.

5.2.5 *Modalités de sorties d'étude*

✓ *Arrêt de participation d'une personne à la recherche*

Le patient pourra retirer son consentement et demander à sortir de l'étude à n'importe quel moment et quelle qu'en soit la raison. Les données recueillies au cours de sa participation à l'étude seront conservées à moins que le patient ne s'y oppose.

L'investigateur pourra interrompre temporairement ou définitivement la participation d'un patient à l'étude pour toute raison qui servirait au mieux les intérêts du patient en particulier en cas d'événements indésirables. Le traitement devra également être suspendu ou arrêté en cas de détérioration significative des échanges gazeux (PaO2/FiO2) qui serait consécutive au traitement en cours par Dornase alfa.

En cas de sortie prématurée, l'investigateur doit en documenter les raisons de façon aussi complète que possible.

En cas de patient perdu de vue, l'investigateur mettra tout en œuvre pour reprendre contact avec la personne afin de connaître les raisons de sa sortie d'essai et son état de santé. Le cahier d'observation devra être rempli jusqu'à la dernière visite effectuée.

✓ *Modalités de suivi des patients sortis d'étude*

La sortie d'étude d'un patient ne changera en rien la prise en charge habituelle par rapport à sa maladie.

✓ *Participation à une autre recherche simultanément ou à la fin de celle-ci*

Les patients inclus dans cette étude ne sont pas autorisés à participer à d'autres recherches à risques (RIPH de type 1) pendant les 8 jours suivant l'inclusion, jusqu'au recueil du critère de jugement principal. Pour éviter toute perte de chance, les patients seront ensuite autorisés à participer à d'autre études de type RIPH 1 pendant la durée de leur suivi (J28 ou sortie d'hospitalisation).

✓ *Arrêt définitif ou temporaire de la recherche*

L'étude pourra être suspendue ou interrompue en cas de survenue d'événements indésirables graves nécessitant une revue du profil d'innocuité du médicament étudié.

De même, des événements imprévus ou de nouvelles informations relatives au médicament étudié, au vu desquels les objectifs de l'étude ne seront vraisemblablement pas atteints, pourront amener le promoteur à interrompre prématurément l'étude.

En cas d'arrêt prématuré de l'étude, le promoteur informera l'ANSM et le CPP dans un délai de 15 jours.

5.2.6 *Recueil des données*

Les données seront collectées dans un cahier d'observation électronique (e-CRF). Toutes les données seront saisies dans l'e-CRF par l'équipe d'investigation de l'étude. Un numéro d'identification unique sera attribué à chaque patient, permettant la pseudonymisation des données. Pour chaque patient, les données directement identifiantes ne seront pas saisies dans l'e-CRF (i.e. seuls le numéro d'inclusion, la date d'inclusion, la première lettre du nom, la première lettre du prénom, le mois et l'année de naissance seront recueillis). Un fichier de correspondance permettant de faire le lien entre la base pseudonymisée et l'identité des patients sera conservé dans le classeur investigateur.

Les données nécessaires à la réponse aux critères de jugement principal et secondaires seront monitorées à intervalles réguliers tout au long de l'essai. Tous les efforts raisonnables devront être faits pour compléter les données le plus tôt possible. L'investigateur principal est responsable de l'exactitude et de l'exhaustivité des données enregistrées dans le cahier d'observation. Après contrôle et validation, la base de données sera gelée et exportée pour analyse.

5.2.7 *Tableau récapitulatif du déroulement de l'étude*

Les éléments notés S correspondent à la pratique usuelle, et les éléments notés R sont ceux ajoutés par la recherche

	**Inclusion** *J0*	**J1 à J7**	**J0, J2, J7, J28 ou sortie**	**J1 à J28**
**Mode de réalisation de la visite**	*Hospitalisation conventionnelle*	*Hospitalisation conventionnelle*	*Hospitalisation conventionnelle*	*Hospitalisation conventionnelle*
Recueil du consentement oral auprès du proche de confiance	R			
Vérification des critères d'inclusion et de non inclusion	R			
Bilan clinique : évaluation clinique sur l'échelle à 7 catégories, évaluation statut vital et ventilatoire	S			R
Calcul du rapport PaO2/FiO2	R			R
Réalisation de l'intervention pour les patients du bras traitement		R		
Prélèvement bronchique pour dosage de la concentration en NETs			R	
Prélèvement sanguin pour dosage des			R	

marqueurs de l'inflammation				
Recueil des événements indésirables				R

5.3 Calendrier de l'étude

**Nombre de patients attendus**	Maximum 100
**Période d'inclusion**	90 jours
**Temps de participation maximum par patient**	28 jours
**Durée prévue jusqu'à la réponse au critère de jugement principal**	7 jours
**Durée totale de l'étude**	4 mois

5.4 Description des mesures prises pour réduire et éviter les biais

La randomisation sera établie selon un ratio 1:1. Les inclusions seront stratifiées sur le centre et sur le rapport PaO2/FiO2 moyen des 4 prélèvements des 24 dernières heures (200 < PaO2/FiO2 ≤ 300 ; 100 < PaO2/FiO2 ≤ 200 ; PaO2/FiO2 ≤ 100).
Les analyses seront effectuées en intention de traiter, l'ensemble des patients randomisés seront inclus dans les analyses selon leur bras de randomisation.

5.5 Bénéfices, risques et contraintes pour le patient participant à la recherche

✓ *Bénéfices*

Les bénéfices individuels attendus sont une amélioration des paramètres respiratoires sous ventilation mécanique avec réduction de l'incidence du SDRA modéré et sévère, une réduction de la durée de ventilation mécanique, une réduction de la durée de séjour en réanimation et à l'hôpital, une réduction de la mortalité à J30.

✓ *Risques*

Risques liés à l'administration de Dornase alfa :

Ils procèdent des effets indésirables de la Dornase alfa, tels que décrits dans le RCP du Pulmozyme®. En effet, même si l'indication retenue dans l'étude (SDRA lié au COVID-19) n'est pas celle de l'AMM (mucoviscidose), les posologies, rythme et voies d'administration seront similaires. Ces effets indésirables sont mineurs et peu fréquents (<1/1000) mais ils ont été décrits dans une population (patients ambulatoires atteints de mucoviscidose) différente de celle des sujets qui participeront à l'étude (patients de réanimation, SDRA lié au COVID-19, sous sédation et ventilation mécanique).
Dans la majorité des cas, les effets indésirables décrits dans le RCP sont modérés et transitoires et ne nécessitent pas de modification de la posologie.

° Affections oculaires : conjonctivite.

° Affections respiratoires, thoraciques et médiastinales : dysphonie, dyspnée, pharyngite, laryngite, rhinites (toutes non infectieuses)

° Affections gastro-intestinales : dyspepsie.

° Affections de la peau et du tissu sous-cutané : rash, urticaire.

° Troubles généraux et anomalies au site d'administration : douleur thoracique (d'origine pleurale/non-cardiaque), fièvre.

° Investigations : diminution de la fonction respiratoire mesurée par les valeurs des explorations fonctionnelles.

La plupart de ces effets indésirables relèvent de signes fonctionnels (dysphonie, dyspepsie, douleur thoracique…) qu'il est impossible de rechercher chez des patients de réanimation sous sédation. La prévention des conjonctivites (occlusion palpébrale soigneuse, application de lubrifiants à base de solution aqueuse sans conservateur et en unidose, type méthylcellulose ou gel visqueux) fait partie des soins courants en réanimation et sera appliquée de façon systématique. L'apparition de réactions anaphylactoïdes de niveau III et IV de la classification de Ring et Messmer conduira à l'interruption du traitement à l'essai, au prélèvement de tubes spécifiques (tryptase sérique, histamine plasmatique) et à la réalisation d'un bilan allergologique à distance de l'événement (3-4 semaines), selon les recommandations en vigueur (SFAR et HAS). Les autres réactions (rash, érythème), donneront lieu à une surveillance clinique simple.

Aucune toxicité systémique de Pulmozyme® n'a été observée, et cela n'est pas attendu dans la mesure ou l'absorption systémique de la Dornase alfa administrée par voie pulmonaire est faible (<2% chez le singe et non quantifiable chez l'homme) et sa demi-vie plasmatique courte (demi-vie d'élimination plasmatique de 3-4 heures chez l'homme).

Le dispositif d'administration (Aerogen Solo®, Aerogen, Irlande, Marquage CE0050) est un dispositif « à patient unique », ce qui rend le risque de contamination croisée quasi-nul. Par ailleurs, les durées d'administration très courtes (5 à 7 minutes par session) et pendant une période également courte (sept jours consécutifs) réduisent massivement le risque hypothétique de prolifération microbienne dans le dispositif et de contamination des voies aériennes du patient. Ces dispositifs sont par ailleurs utilisés couramment et sont donc bien connus des soignants des services de réanimation.

Risques liés aux prélèvements plasmatiques supplémentaires

Le risque théorique d'anémie et d'exposition indue à la transfusion sanguine provoqués par le prélèvement supplémentaire de sang au cours des cinq premiers jours est négligeable. Et ceci notamment au regard des prélèvements sanguins réalisés pour la conduite des soins courants. Le volume a été choisi afin d'obtenir le meilleur compromis entre une spoliation sanguine réduite au minimum et des prélèvements qui restent exploitables pour la détermination des concentrations plasmatiques des biomarqueurs investigués. Tous les patients admis en réanimation sont équipés d'un cathéter artériel, posé sous anesthésie générale dès l'admission. Les prélèvements sanguins successifs seront réalisés via ce cathéter, sans nécessité de ponctions multiples, réduisant à néant l'inconfort lié aux prélèvements sanguins itératifs.

6 Description des médicaments de l’étude

6.1 Identification du traitement

Dornase alfa (Pulmozyme®, Résumé des Caractéristiques du Produit MAJ du 19/12/2017), Appartenance au groupe R de la classification ATC : SYSTEME RESPIRATOIRE (R05CB13) et Pneumologie dans la classification pharmacothérapeutique. Médicament de Liste I soumis à une prescription initiale hospitalière semestrielle. Renouvellement non restreint.
AMM dornase alpha (Pulmozyme®) dans la mucoviscidose : 10 mars 1994
Composition : dornase alfa (2500 UI/2,5mL, correspondant à 1000 U/mL (soit 1 mg/mL) ; 1 unité Genentech/mL = 1 µg/mL). Excipients : chlorure de sodium, chlorure de calcium dihydraté, eau pour préparation injectable. La dornase alfa est une désoxyribonucléase de type 1 : protéine humaine glycosylée et phosphorylée produite dans une lignée cellulaire d'ovaire d'hamster chinois CHO A14.16-1 MSB # 757 par la technique de l'ADN recombinant.
Pulmozyme® peut également être utilisé avec un système nébuliseur-réutilisable/compresseur pneumatique tel que Pari LL/PariBoy, Pari LC/PariBoy ou Master, Aiolos/Aiolos, Side Stream/CR50 ou Mobil Aire ou Porta-Neb.
Les nébuliseurs ultrasoniques peuvent ne pas être adaptés à l'administration de Pulmozyme® en raison du risque d'inactivation de Pulmozyme® et des caractéristiques d'administration inadaptées de l'aérosol délivré.
Le mode d'emploi fourni par les fabricants concernant l'utilisation et l'entretien des systèmes des nébuliseurs et compresseurs doit être suivi.
L'usage de tente de nébulisation n'est pas nécessaire.
Les ampoules ne doivent être utilisées que pour une seule administration. Toute solution non utilisée ou à jeter doit être éliminée conformément à la réglementation locale en vigueur.

6.2 Conditionnement et étiquetage

Il s’agit d’une étude ouverte. Le conditionnement du médicament ne sera pas modifié. Le circuit et les numéros de lot devront être tracés. Un contre-étiquetage sera réalisé par un pharmacien dans chacune des PUI. Le pharmacien s’engagera à ce que cet étiquetage n’occulte pas les mentions originales du produit commercial.

6.3 Fabrication et distribution des traitements

Le produit actif à l’essai (dornase alfa) sera acheté auprès du laboratoire qui commercialise le Pulmozyme® (Roche)

La traçabilité des traitements expérimentaux sera assurée conformément à la règlementation des essais cliniques. Le circuit des traitements, les documents de traçabilité et les modalités de distribution et d'administration seront répertoriés dans les Procédures Circuit Pharmaceutique.

6.4 Administration

Posologie et mode d'administration dans l'étude COVIDornase :

Chez des patients de réanimation (sous sédation et ventilation mécanique), le mode d'administration préconisé chez des sujets mucoviscidosiques conscients et en ventilation spontanée (système nébuliseur/compresseur pneumatique) est remplacé par un nébuliseur à tamis vibrant, permettant l'administration de dornase alfa sans participation du patient, directement sur le circuit respiratoire

Des études expérimentales et cliniques ont démontré que l'administration de dornase alfa par nébuliseur à tamis vibrant accélérait la vitesse d'administration, favorisait la biodisponibilité pulmonaire sans dénaturer le produit actif ni altérer ses propriétés physico-chimiques. Nous avons fait le choix de ce mode d'administration et préconisé l'utilisation de nébuliseurs à tamis Aerogen solo® (Marquage CE 0050), distribués par la firme Aerogen et utilisés quotidiennement en réanimation pour l'administration d'autres agents thérapeutiques (bronchodilatateurs, antibiotiques…) chez des patients ventilés et qui sont donc bien connus des soignants.

Justification de la dose choisie pour la recherche :

La posologie (2500 UI par administration, sept jours consécutifs à 12h d'intervalle) a été choisie compte tenu de ce qui est connu de la physiopathologie des NETs dans les pneumonies virales graves ainsi que la pharmacocinétique de la dornase alfa et sa biodisponibilité pulmonaire. En effet, la formation intrapulmonaire des NETs est un phénomène extrêmement rapide, du moins chez l'animal[14] et dans un faible collectif de patients en réanimation pour traumatisme grave.[15] Cette temporalité justifie selon nous un schéma d'administration simple sur une durée courte à l'aide d'un dispositif qui optimise la biodisponibilité pulmonaire de la dornase alfa.

Il s'agit donc bien dans le cas présent d'une utilisation hors AMM de la dornase alfa mais, compte tenu d'un rationnel physiopathologique étayé, de résultats pré-cliniques encourageants, de l'excellent profil de tolérance de ce médicament commercialisé depuis 1994 et des modalités d'administration choisies qui sont conformes au RCP actuelles du produit, cette utilisation nous semble justifiée dans le cadre d'une étude clinique prospective chez des patients qui bénéficieront du niveau de surveillance le plus élevé (réanimation) dans notre établissement de santé.

Période de suivi après traitement :

Compte tenu d'une administration brève (sur sept jours), il n'y aura pas de suivi au-delà des 30 jours prévus par l'étude.

7 Evaluation de la sécurité

7.1 Définitions

Évènement indésirable : toute manifestation nocive survenant chez une personne qui se prête à une recherche impliquant la personne humaine que cette manifestation soit liée ou non à la recherche ou au produit sur lequel porte cette recherche.

Effet indésirable : toute réaction nocive et non désirée à un médicament expérimental quelle que soit la dose administrée.

Évènement (ou/effet) indésirable grave (EIG) : tout évènement (ou effet) indésirable qui :

- entraîne la mort,
- met en jeu le pronostic vital de la personne qui se prête à la recherche,
- nécessite une hospitalisation ou la prolongation d'une l'hospitalisation,
- provoque une incapacité ou un handicap important ou durable,
- se traduit par une anomalie ou une malformation congénitale (exposition intra utero)
- *ainsi que tout évènement (ou effet) considéré comme médicalement significatif (c'est à dire ayant des conséquences cliniques importantes mais ne correspondant pas à l'un des autres critères de gravité).*

Et s'agissant du médicament, quelle que soit la dose administrée.

N'est pas considéré comme EIG :

- o Une hospitalisation pour une procédure médicale/chirurgicale prévue avant l'inclusion ou prévue dans le protocole
- o Une hospitalisation pour une pathologie présente avant l'inclusion et ne s'aggravant pas en cours d'étude
- o Une circonstance de vie n'ayant aucune incidence sur l'état de santé et ne nécessitant aucune intervention médicale/chirurgicale (ex : prolongation d'hospitalisation en attendant une place dans un autre service ou établissement, hospitalisation pour cause d'aidants indisponibles, etc…)

Effet indésirable inattendu : tout effet indésirable du produit dont la nature, la sévérité, la fréquence ou l'évolution ne concorde pas avec les informations de référence sur la sécurité mentionnées dans le résumé des caractéristiques du produit ou dans la brochure pour l'investigateur lorsque le produit n'est pas autorisé.

Lien de causalité : relation entre l'événement indésirable et le médicament (ou l'étude). Les facteurs à prendre en compte pour la détermination de l'imputabilité sont : les critères chronologiques (délai de survenue de l'effet indésirable, évolution de l'effet indésirable à l'arrêt du traitement, réapparition à la reprise), et les critères sémiologiques (mécanisme d'action ou explication pharmacodynamique,

facteurs favorisants ou antécédents similaires, diagnostics différentiels possibles, examens complémentaires prouvant la cause).

- *non lié* : l'évènement est clairement lié à d'autres causes comme *non lié* : l'évènement est clairement lié à d'autres causes comme une progression de la maladie, un traitement concomitant, une maladie intermittente
- *possiblement lié* : évènement clinique ou biologique avec une relation chronologique et sémiologique compatible

Intensité : l'intensité des événements est évaluée par l'investigateur selon la classification suivante : Grade 1 : Léger ; asymptomatique ou symptômes légers ; diagnostic à l'examen clinique uniquement ; ne nécessitant pas de traitement - Grade 2 : Modéré ; nécessitant un traitement minimal, local ou non-invasif ; interférant avec les activités instrumentales de la vie quotidienne - Grade 3 : Sévère ou médicalement significatif mais sans mise en jeu immédiate du pronostic vital ; indication d'hospitalisation ou de prolongation d'hospitalisation ; invalidant ; interférant avec les activités élémentaires de la vie quotidienne - Grade 4 : Mise en jeu du pronostic vital ; nécessitant une prise en charge en urgence - Grade 5 : Décès lié à l'EI

Incident : Fait susceptible d'affecter la qualité et la sécurité d'emploi du produit et donc de représenter un risque pour la santé des personnes. Cet incident peut survenir au cours de la chaîne de fabrication du produit de santé et jusqu'à son utilisation. Il est susceptible d'entraîner un effet indésirable.

Incident grave : Incident susceptible d'entraîner des effets indésirables graves.

Fait nouveau : Il s'agit de toute nouvelle donnée pouvant conduire à :

- ✓ une réévaluation du rapport des bénéfices et des risques de la recherche ou du produit objet de la recherche
- ✓ des modifications dans l'utilisation de ce produit, dans la conduite de la recherche, ou des documents relatifs à la recherche
- ✓ ou à suspendre ou interrompre ou modifier le protocole de la recherche ou des recherches similaires. Pour les essais portant sur la première administration ou utilisation d'un produit de santé chez des personnes qui ne présentent aucune affection : tout effet indésirable grave.

Un fait nouveau peut également correspondre à une suspicion d'effet indésirable grave inattendu *(SUSAR*).

Pour les essais portant sur la première administration ou utilisation d'un produit de santé chez des personnes qui ne présentent aucune affection : tout effet indésirable grave est considéré comme un fait nouveau.

7.2 Enregistrement et notification des évènements indésirables par l'investigateur

7.2.1 *Evènements indésirables*

L'investigateur reporte les évènements indésirables dans le cahier d'observation.
Il évalue pour chaque évènement indésirable, son intensité, sa gravité et le lien de causalité avec la procédure/la technique étudiée (ou comparative) ou avec les autres traitements éventuels.

7.2.2 *Evènements indésirables graves*

L'investigateur notifie **sans délai dès qu'il en a connaissance** les événements indésirables graves (EIG) au promoteur, à l'aide du « formulaire de notification d'EIG ».
L'évolution clinique ainsi que les résultats des éventuels bilans cliniques et des examens diagnostiques et/ou de laboratoire, ou toute autre information permettant une analyse adéquate du lien de causalité de l'EIG sont à préciser :
- soit sur la notification initiale d'EIG s'ils sont immédiatement disponibles,
- soit ultérieurement et le plus rapidement possible, sur un nouveau « formulaire de notification d'EIG » de suivi (le 1er suivi à adresser dans les 8 jours après la notification initiale).

La notification initiale, et, le cas échéant, ses rapports de suivi, sont transmis par email ou par télécopie au promoteur :

Vigilance des Essais Cliniques, Service de Recherche Clinique

Fondation A. de Rothschild (Paris)

Mail : shoudas@for.paris

Télécopie : 01 48 03 64 30

L'investigateur communique au promoteur tous les renseignements complémentaires demandés (compte-rendu d'examens complémentaires, compte-rendu d'hospitalisation, résultats d'autopsie, etc.).
Chaque document transmis par l'investigateur devra comporter l'acronyme de la recherche et l'identifiant du patient attribué dans le cadre de la recherche. L'original des documents transmis est conservé sur le site d'investigation.
Tout patient présentant un EIG recevra la prise en charge adaptée à son état et sera suivi jusqu'à la résolution de l'événement ou à un retour à l'état antérieur. Si cela s'avère nécessaire, le médicament expérimental sera arrêté.

Tout EIG doit être notifié au promoteur :

- à partir de la date de signature du consentement
- jusqu'à la fin de suivi du sujet,

mais également sans limitation de temps, lorsque l'EIG est susceptible d'être dû au médicament.
Dans le cadre de cette recherche, l'investigateur se référera au paragraphe 7.2.3.

7.2.3 *Evènements indésirables d'intérêt particulier*

L'investigateur notifie **sans délai dès qu'il en a connaissance** au promoteur, les EI suivants à l'aide du « formulaire de notification d'EIG » car ils ont été identifiés comme nécessitant une surveillance tout au long de la recherche *(s'ils ne répondent pas à la définition des EIG, ils seront classés comme « médicalement significatifs)* :

<u>Particularités pour cette recherche :</u>
Compte tenu du fait que d'une part, les participants à la recherche sont susceptibles de présenter un nombre important d'évènements indésirables graves liés à leur prise en charge en réanimation, les évènements indésirables graves à notifier au promoteur seront les suivants :

- Réactions allergiques de grade 3 ou 4 ;
- Aggravation majeure des paramètres ventilatoires dans l'heure suivant l'administration du médicament avec nécessité d'une ré-intubation : Dans le cadre de l'administration de dornase alpha, nous considérerons comme effet indésirable grave la nécessité dans l'heure suivant l'administration, d'extuber le patient pour cause d'obstruction de la sonde d'intubation, et de le ré-intuber ensuite.
- Tout évènement indésirable que l'investigateur souhaiterait signaler ;
- Toute évolution fatale en précisant la cause.

7.3 Prise en charge des évènements indésirables graves par le promoteur

7.3.1 *Evaluation médicale*

Pour chaque EIG, le promoteur (le vigilant) évalue de son côté la gravité et le lien avec le médicament, il juge du caractère attendu (listé) ou inattendu selon le document de référence.
Il sollicite l'investigateur (ou l'ARC/TEC de l'étude), pour obtenir les informations complémentaires sur les circonstances de survenue de l'EIG, son traitement et son évolution.
Il code l'EIG selon la méthode MedDRA et rédige le narratif de l'EIG en anglais

7.3.2 *Déclaration des suspicions d'effets indésirables graves inattendus (SUSAR)*

Le promoteur déclare à l'ANSM toute suspicion d'effet indésirable grave inattendu (SUSAR : *suspected unexpected serious adverse reaction*) :

- En cas de décès ou de mise en jeu du pronostic vital : sans délai à compter du jour où il en a connaissance
- Dans les autres cas ; au plus tard dans un délai de 15 jours à compter du jour où il en a connaissance

Les informations complémentaires pertinentes doivent être adressées dans les 8 jours.

Le promoteur informe les investigateurs de l'étude des suspicions d'effets indésirables graves inattendus qui pourraient avoir un impact défavorable sur la sécurité des personnes qui se prêtent à la recherche.

7.4 Fait nouveau et mesures urgentes de sécurité

En cas de survenue d'un fait nouveau durant l'étude, le promoteur en informe sans délai, l'ANSM et le CPP en précisant les éventuelles mesures de sécurité prises (*Si le protocole prévoit l'inclusion de volontaires sains, le promoteur déclarera tout fait nouveau également à l'ARS).*
Les informations complémentaires pertinentes doivent être adressées dans les 8 jours.

De plus, en cas :

- D'arrêt de la recherche : le promoteur déclare la fin de la recherche dans un délai de 15 jours
- De modification substantielle : le promoteur dépose une demande de modification substantielle dans un délai de 15 jours.

7.5 Rapport annuel de sécurité

Habituellement, le promoteur rédige, un rapport annuel de sécurité relatif à l'essai clinique concerné. Ce rapport est adressé l'ANSM et au CPP dans les 60 jours qui suivent la date anniversaire de la recherche, qui est la date d'autorisation de l'ANSM.
Dans le cas de cette recherche prévue sur quelques mois, le promoteur rédigera un rapport de sécurité ayant comme date de fin, le dernier suivi du dernier patient inclus.

7.6 Comité de surveillance indépendant

Un comité de surveillance indépendant sera mis en place pour cette étude. Il inclura un méthodologiste, un réanimateur et un infectiologue, tous trois indépendants de l'étude et n'exerçant pas au sein des centres investigateurs. Il se réunira lorsque 10 patients par bras auront été inclus et suivis jusqu'à 7 jours (durée de l'intervention) afin de statuer sur la sécurité de l'essai. Une première analyse intermédiaire de sécurité a été prévue à ce stade (cf section 8.2.6). Si l'essai est poursuivi, le CSI se réunira également lorsque 25 patients par bras auront été inclus et suivis jusqu'à 7 jours. Une seconde analyse intermédiaire de sécurité a été prévue à ce stade (cf section 8.2.6). Le cas échéant le CSI pourra aussi être saisi à la demande du promoteur. Une charte de fonctionnement a été rédigée concernant le fonctionnement de ce comité.

8 Analyses statistiques

8.1 Calcul du nombre de sujets

Il s'agit d'un étude pilote pour laquelle nous ne disposons pas des hypothèses nécessaires à la réalisation d'un calcul de nombre de sujets à inclure En tenant compte du recrutement potentiel des centres, nous estimons le potentiel d'inclusion à 100 patients maximum soit 50 patients/bras au maximum.

8.2 Description des analyses statistiques utilisées

L'analyse statistique sera réalisée sous la responsabilité du Service de Recherche Clinique de la Fondation Adolphe de Rothschild.

8.2.1 *Choix des personnes à inclure dans les analyses*

Tous les patients inclus seront analysés. Les analyses seront réalisées en Intention de Traiter (ITT).

8.2.2 *Statistiques descriptives*

Un diagramme de flux établi selon les recommandations CONSORT résumera le nombre de patients screenés, inclus, randomisés, perdus de vue et analysés pour chaque bras de randomisation. Les raisons de non-participation à chaque étape seront décrites.

Une analyse descriptive des données sera réalisée. Cette analyse comportera des estimations ponctuelles, nombres et pourcentages pour les variables qualitatives, moyenne, écart-type, médiane et range pour les variables quantitatives. La normalité des variables continues sera évaluée graphiquement et à l'aide d'un test de normalité (e.g. Shapiro-Wilk). Une description des données manquantes de chaque variable (effectif et pourcentage) sera réalisée.

8.2.3 *Comparaisons des groupes à la baseline*

La comparabilité à la baseline entre le bras expérimental et le bras contrôle sera évaluée au moyen d'un test t de Student (ou test de Mann-Whitney si nécessaire) pour les paramètres continus et d'un test de Chi^2 (ou test exact de Fisher si nécessaire) pour les paramètres qualitatifs.

8.2.4 *Analyse du critère de jugement principal*

La comparaison entre les groupes sera réalisée à l'aide d'une régression logistique ajustée sur les facteurs de stratification et sur l'administration concomitante de traitements anti-infectieux et anti-inflammatoires.

8.2.5 *Analyse des critères de jugement secondaires*

Le taux de mortalité toutes causes à J28, le taux de patients ayant présenté une évolution clinique favorable à J28, et le taux d'EIG seront analysés à l'aide de régressions logistiques ajustées sur les facteurs de stratification et sur l'administration concomitante de traitements anti-infectieux et anti-inflammatoires.

Les nombres de jours vivant sans ventilation mécanique et hors réanimation, seront analysés à l'aide de régressions linéaires ajustées sur les facteurs de stratification et sur l'administration concomitante de traitements anti-infectieux et anti-inflammatoires.

Les délais jusqu'à l'amélioration du SDRA (selon CJP) et jusqu'à sortie de la réanimation seront analysés par des modèles de Cox ajustés sur les facteurs de stratification et sur l'administration concomitante de traitements anti-infectieux et anti-inflammatoires.

Les concentrations en NETs aux différents temps de prélèvement et les concentrations des différents marqueurs de l'inflammation aux différents temps seront analysés graphiquement et des analyses exploratoires comparatives pourront être réalisées.

8.2.6 *Analyses intermédiaires de sécurité*

Pour des raisons éthiques, compte tenu de l'absence d'expérience avec l'administration de Pulmozyme dans le SDRA a fortiori lié à COVID-19, des inconnus de son effet sur un tissu pulmonaire lésé en rapport avec la virose, et du contexte de l'étude où le pronostic vital est engagé à court terme, deux analyses intermédiaires sont prévues et seront présentées au CSI afin d'évaluer précocement le possible effet délétère de ce traitement.

Ces analyses seront basées sur le critère de jugement principal binaire, à savoir l'amélioration d'au moins 1 point sur l'échelle de Berlin entre le jour 7 et la randomisation.

Soient T et C les bras traitement et contrôle, et Y le critère de jugement principal. Soit $p_{.} = P(Y = 1|.)$, la proportion d'amélioration dans chaque bras. On choisit comme distribution a priori pour $p_{.}$ une distribution non informative beta(1,1). Il s'agit d'un choix classique de distribution a priori utilisée pour l'estimation d'une proportion, qui peut être conjuguée à une distribution binomiale et correspond à un a priori non informatif uniforme pour toutes les proportions de réponses possibles.

La différence entre l'effet dans les bras traitement T et contrôle C ,$\Delta = p_T - p_C$ est le paramètre d'intérêt de cette analyse

Au moment de l'analyse intermédiaire, les proportions d'amélioration pour chaque bras de l'essai avec leur intervalle de crédibilité à 95% seront présentés, ainsi que la différence Δ entre les proportions d'amélioration dans les bras traitement et contrôle, et son intervalle de crédibilité à 95%. Dans un contexte Bayésien, les décisions de poursuites de l'essai seront basées sur les probabilités a posteriori suivantes :

- P1 = P(Δ > 0 | data)   effet bénéfique

- P2 = P(Δ > 0.2 | data) effet bénéfique cliniquement important
- P3 = P(Δ < 0 | data) inefficacité ou effet délétère
- P4 = P(Δ < -0.2 | data) effet délétère important

L'objectif de ces analyses est de pouvoir stopper précocement l'essai en cas de signal d'absence d'effet du traitement (futilité) ou d'effet délétère du traitement (toxicité). Dans ce but, les règles de décision suivantes sont proposées :

1. Arrêt avec preuve d'inefficacité si P3 > 0.8
2. Arrêt avec preuve d'effet délétère si P4 > 0.67

Il est à noter que la règle (2) ne peut survenir que dans des situations où la règle (1) est également survenue (cf figure 1).

Deux analyses intermédiaires sont planifiées : la première après l'inclusion de 10 patients dans chaque bras, et la seconde après l'inclusion de 25 patients dans chaque bras.

Figure 1 : règles d'arrêt pour les deux analyses intermédiaires

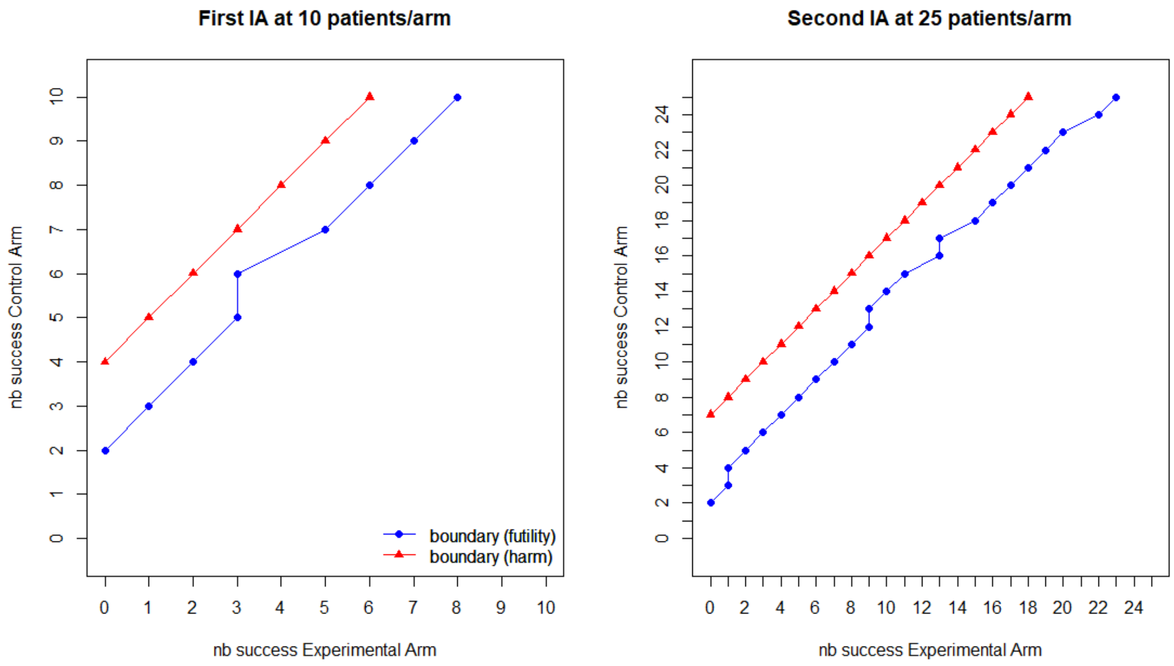

8.3 Niveau de significativité statistique

Risque alpha fixé à 0.05.

8.4 Modalités de prise en compte des données manquantes, non utilisées ou non valides

A priori, les patients étant hospitalisés, les données manquantes devraient être très limitées. En cas de données manquantes, les données ne seront pas remplacées mais une analyse de sensibilité avec une imputation par LOCF sera réalisée.

8.5 Gestion des modifications apportées au plan statistique initial

Des modifications à la stratégie initiale pourront être apportées en fonction de l'avancement de l'étude et reprises dans le plan d'analyse statistique qui sera rédigé juste avant le gel de la base de données. Les éventuelles modifications à apporter au plan d'analyse statistique seront proposées par le méthodologiste de l'étude et validées par consensus avec le statisticien et l'investigateur principal.

9 Dispositions réglementaires

La Fondation Adolphe de Rothschild est promoteur de cette **recherche impliquant la personne humaine, de type interventionnelle**, selon l'article L1121-1 1er alinéa du Code de la Santé Publique, modifié par Ordonnance n°2016-800 du 16 juin 2016 (art. 1).

9.1 Demande d'autorisation auprès de l'autorité compétente (Agence Nationale de Sécurité du Médicament et des produits de santé - ANSM)

Le présent protocole fera l'objet d'une demande d'autorisation auprès de l'autorité compétente (ANSM). L'autorité compétente, définie à l'article L. 1123-12, modifié par Ordonnance n°2016-800 du 16 juin 2016, se prononce au regard de la sécurité des personnes qui se prêtent à une recherche interventionnelle, en considérant notamment la sûreté et la qualité des produits utilisés au cours de la recherche conformément, le cas échéant, aux référentiels en vigueur, leur condition d'utilisation et la sécurité des personnes au regard des actes pratiqués et des méthodes utilisées ainsi que les modalités prévues pour le suivi des personnes. Elle se prononce en outre sur la pertinence de la recherche, le caractère satisfaisant de l'évaluation des bénéfices et risques attendus et le bien-fondé des conclusions.

9.2 Demande d'avis auprès du Comité de Protection des Personnes (CPP)

En accord avec l'article L.1123-6 du Code de Santé Publique modifié par Ordonnance n°2016-800 du 16 juin 2016 (art 3.), cette recherche impliquant la personne humaine est soumise à l'avis d'un CPP désigné de manière aléatoire, sans lequel elle ne pourra débuter. L'avis de ce comité sera notifié à l'autorité compétente par le promoteur avant le démarrage de la recherche.

9.3 Engagements – aspects éthiques

Le promoteur et les investigateurs s'engagent à ce que cette recherche soit réalisée en conformité avec les dispositions législatives en vigueur, les Bonnes Pratiques Cliniques et la déclaration d'Helsinki.

Les investigateurs s'engagent à respecter le protocole en tout point en particulier en ce qui concerne le recueil du consentement et la notification et le suivi des événements indésirables graves. Un exemplaire de l'engagement de responsabilités daté et signé par les investigateurs sera remis au représentant du promoteur.

9.4 Déclaration du démarrage et de la fin de l'étude

Dès la première inclusion, le promoteur informera sans délai l'ANSM et le CPP de la date effective de démarrage de l'étude. La date effective de démarrage correspond à la date de signature du consentement par la première personne qui se prête à la recherche.
La date de fin d'étude sera transmise par le promoteur à l'ANSM et au CPP dans un délai de 90 jours. La date de fin de la recherche correspond au terme de la participation de la dernière personne qui se prête à la recherche, ou le cas échéant, au terme défini dans le protocole.

9.5 Note d'information et recueil du consentement écrit du patient

Une note d'information précisant les informations à fournir selon l'article 13 du Règlement Général sur la Protection des Données de l'Union européenne (RGPD) et soumise préalablement au CPP, sera expliquée et remise aux patients ou à la personne de confiance. Le détail de la procédure d'information est mentionné plus haut dans ce protocole.
L'information donnée aux personnes et le recueil de leur consentement doivent être notés et datés dans le dossier médical du patient. En cas de refus de participation, le patient bénéficiera de la prise en charge habituelle. Lorsque cette recherche sera terminée, la personne qui se prête à la recherche pourra être informés des résultats globaux selon les modalités qui seront précisées dans le document d'information. Les informations relatives aux droits des personnes participant à cette recherche (droit d'accès et de rectification, droit d'opposition à la transmission des données couvertes par le secret professionnel susceptibles d'être utilisées dans le cadre de cette recherche) sont intégrées dans les notes d'information.

9.6 Modifications du protocole

L'investigateur coordonnateur (ou principal en cas d'étude monocentrique) informera le promoteur de tout projet de modification du protocole.

Le promoteur est seul autorisé à modifier le protocole et décidera si la modification envisagée relève ou non d'une modification substantielle. La demande de modification sera adressée par le promoteur à l'ANSM et/ou au CPP pour autorisation et avis selon les cas.

On entend par modifications substantielle : les modifications qui ont un impact significatif sur tout aspect de la recherche, notamment sur la protection des personnes, y compris à l'égard de leur sécurité, sur les conditions de validité de la recherche, le cas échéant sur la qualité et la sécurité des produits expérimentés, sur l'interprétation des documents scientifiques qui viennent appuyer le déroulement de la recherche ou sur les modalités de conduite de celle-ci. Une modification non substantielle du protocole est une modification mineure ou une clarification sans retentissement sur la conduite de

l'essai.

Dès réception de l'avis et/ou de l'autorisation, la version amendée du protocole sera alors transmise à tous les investigateurs par le promoteur.

9.7 Déclaration CNIL (Commission Nationale de l'Informatique et des Libertés)

Cette recherche est soumise à la loi n°78-17 du 6 janvier 1978 relative à l'informatique, aux fichiers et aux libertés modifié par la loi n°2018-493 du 20 juin 2018 relative à la protection des données personnelles, ainsi qu'au Règlement Général sur la Protection des Données de l'Union européenne (RGPD). Dans le cadre du traitement de données de santé à des fins de recherche scientifique, cette recherche est soumise à l'article 9 du RGPD, alinéas I et J de l'article 9.2.

Une méthodologie de référence spécifique au traitement de données personnelles opéré dans le cadre des recherches impliquant la personne humaine, de type interventionnelle, a été établie par la CNIL (MR-001). Cette méthodologie permet une procédure de déclaration simplifiée lorsque la nature des données recueillies dans la recherche et leur traitement sont compatibles avec le document de référence de la CNIL. Les données recueillies au cours de l'étude ne mentionneront ni le nom ni le prénom des patients inclus, mais seront codées. Les renseignements obtenus seront sauvegardés sur fichier informatique. Seules les personnes directement impliquées dans l'étude seront habilitées à intervenir sur ces fichiers. Les informations relatives aux droits des personnes participant à cette recherche (droit d'accès et de rectification, droit d'opposition à la transmission des données couvertes par le secret professionnel susceptibles d'être utilisées dans le cadre de cette recherche) seront intégrées dans les notes d'information.

9.8 Droits d'accès aux données et documents sources

Toutes les données et informations concernant le patient resteront strictement confidentielles. Les personnes ayant un accès direct conformément aux dispositions législatives et réglementaires en vigueur, notamment les articles L.1121-3 et R.5121-13 du Code de la Santé Publique (par exemple, les investigateurs, les personnes chargées du contrôle de qualité, les moniteurs, les assistants de recherche clinique, les auditeurs et toutes personnes appelées à collaborer aux essais) prendront toutes les précautions nécessaires en vue d'assurer la confidentialité des informations relatives au médicament expérimental, aux essais, aux personnes qui s'y prêtent et notamment en ce qui concerne leur identité ainsi qu'aux résultats obtenus. Les données collectées par ces personnes au cours des contrôles de qualité ou des audits seront alors rendues anonymes.

L'investigateur garantira l'accès aux données sources pour le moniteur, l'auditeur ou l'inspecteur de l'autorité administrative compétente. Il s'engage à accepter les contrôles du promoteur et à fournir l'accès aux données sources (dossiers médicaux, fichiers informatiques, documents de l'étude,…).

Des visites de monitoring seront effectuées dans chaque centre selon une fréquence définie par le promoteur. Lors de ces visites sur site en accord avec l'investigateur, les éléments suivants sont revus :
- respect du protocole et des procédures qui y sont rattachées,
- assurance qualité des données recueillies dans le cahier d'observation : exactitude, données manquantes, cohérence des données, contrôle des documents sources.

Selon les articles 17.3.c et 17.3.d. du RGPD, en cas de retrait du consentement, et d'une demande d'effacement des données, les données recueillies préalablement au retrait du consentement pourront ne pas être effacées et pourront continuer à être traitées dans les conditions prévues par la recherche, si l'effacement de ces données est susceptible de rendre impossible ou de compromettre gravement la réalisation des objectifs de la recherche.

Les participants ont un droit de limitation de traitement de leurs données personnelles selon les conditions décrites à l'article 18 du RGPD.

Les données pourront être consultées uniquement par le personnel autorisé dans le cadre d'un contrôle qualité.

9.9 Archivage

Les documents et données relatifs à cette recherche seront archivés par l'investigateur et par le promoteur, pour une durée de 15 ans après la fin de la recherche.
Cet archivage indexé comporte :
- Les copies de l'autorisation de l'ANSM
- Les copies de l'avis du CPP
- Les versions successives du protocole (identifiées par le n° de version et la date de version)
- Les courriers de correspondance entre promoteur et les investigateurs
- Le cahier d'observation complété et validé de chaque sujet inclus
- Les formulaires de consentement
- Toutes les annexes spécifiques à l'étude (notamment le CV des investigateurs)
- Le rapport final de l'étude provenant de l'analyse statistique et du contrôle qualité de l'étude (double transmis au promoteur)
- Les certificats d'audits éventuels réalisés au cours de la recherche

- La base de données ayant donné lieu à l'analyse statistique, devant aussi faire l'objet d'archivage par le responsable de l'analyse (support papier ou informatique)

10 Assurance et financement

10.1 Assurance

Dans cette recherche impliquant la personne humaine, de type interventionnelle, la Fondation Adolphe de Rothschild, en tant que promoteur, contractera auprès de la SHAM une police d'assurance pour toute la durée de la recherche. Cette assurance garantira sa propre responsabilité civile ainsi que celle de tout intervenant (médecin ou personnel impliqué dans la réalisation de la recherche), indépendamment de la nature des liens existant entre les intervenants et le promoteur (Article L1121-10 du Code de la Santé Publique, modifié par loi n°2012-300 du 5 mars 2012 – art. 1 et article R 1121-4, modifié par décret n°2106-1537 du 16 novembre 2016 – art.3).

10.2 Financement

Une demande de financement par un PHRC National (COVID-19) a été réalisée pour cette étude.
Le promoteur prend en charge les frais supplémentaires liés à d'éventuels fournitures ou examens spécifiquement requis par le protocole de la recherche pour la mise en œuvre de celui-ci. Lorsque la recherche est réalisée dans un établissement de santé, la prise en charge de ces frais fait l'objet d'une convention entre le promoteur et le représentant légal de cet établissement.

11 Règles relatives à la publication et rapport final

11.1 Enregistrement de l'étude

L'étude sera enregistrée sur un site web en libre accès (https://clinicaltrials.gov/) avant l'inclusion du premier patient.

11.2 Rapport final

Le rapport d'étude final mentionné à l'article R.1123-67 du Code de la Santé Publique sera rédigé et signé par le promoteur et l'investigateur. Les résultats de l'étude seront transmis dans la base EudraCT dans un délai d'un an suivant la fin de l'essai, c'est-à-dire au terme de la participation de la dernière personne qui se prête à la recherche.

11.3 Communication et publication des résultats

Conformément à l'article R 5121-13 du Code de la Santé Publique, les essais ne peuvent faire l'objet d'aucun commentaire écrit ou oral sans l'accord conjoint de l'investigateur et du promoteur.

La Fondation Adolphe de Rothschild, promoteur de l'étude, est propriétaire des données et des résultats de l'étude. Aucune utilisation ou transmission à un tiers ne peut être effectuée sans son accord préalable. Les découvertes résultant de l'essai deviendront automatiquement la propriété exclusive du promoteur qui pourra utiliser ces informations de la manière qu'il jugera convenable.

Les communications et publications des résultats de l'étude seront réalisées sous la responsabilité de l'investigateur coordonnateur avec l'accord des investigateurs responsables et des autres coauteurs associés. Elles devront décrire de façon honnête et équilibrée tous les aspects de l'étude sans tenir compte d'autres intérêts, notamment non scientifiques. Les coauteurs des publications seront les investigateurs et les cliniciens impliqués (au prorata de leur contribution), le méthodologiste et le biostatisticien référent de l'étude, et les chercheurs associés (à définir selon le cas). Les autres membres du personnel du service de recherche clinique ayant activement participé à l'élaboration, au déroulement du protocole et à la rédaction des résultats devront également être cités. Les règles de publications suivront les recommandations du réseau EQUATOR (https://www.equator-network.org/reporting-guidelines/). La Fondation Adolphe de Rothschild sera mentionnée comme étant le promoteur de la recherche. Cette dernière a la maîtrise de la première publication.

Toute requête pour cacher des résultats, changer et atténuer le contenu du rapport final ou des publications sera systématiquement rejetée par le promoteur. L'investigateur adressera une copie des publications au promoteur.

12 Références

1. Guan W, Ni Z, Hu Y, Liang W, Ou C, He J, et al. Clinical Characteristics of Coronavirus Disease 2019 in China. *N. Engl. J. Med.* 2020;NEJMoa2002032.
2. Acute Respiratory Distress Syndrome: The Berlin Definition. *JAMA* [Internet]. 2012 [cited 2020 Mar 25];307. Available from: http://jama.jamanetwork.com/article.aspx?doi=10.1001/jama.2012.5669
3. Brinkmann V. Neutrophil Extracellular Traps Kill Bacteria. *Science*. 2004;303:1532–1535.
4. Tate MD, Brooks AG, Reading PC. The role of neutrophils in the upper and lower respiratory tract during influenza virus infection of mice. *Respir. Res.* 2008;9:57.
5. Narasaraju T, Yang E, Samy RP, Ng HH, Poh WP, Liew A-A, et al. Excessive Neutrophils and Neutrophil Extracellular Traps Contribute to Acute Lung Injury of Influenza Pneumonitis. *Am. J. Pathol.* 2011;179:199–210.
6. Porto BN, Stein RT. Neutrophil Extracellular Traps in Pulmonary Diseases: Too Much of a Good Thing? *Front. Immunol.* [Internet]. 2016 [cited 2020 Mar 23];7. Available from: http://journal.frontiersin.org/Article/10.3389/fimmu.2016.00311/abstract
7. Kolaczkowska E, Jenne CN, Surewaard BGJ, Thanabalasuriar A, Lee W-Y, Sanz M-J, et al. Molecular mechanisms of NET formation and degradation revealed by intravital imaging in the liver vasculature. *Nat. Commun.* 2015;6:6673.
8. Dubois AV, Gauthier A, Bréa D, Varaigne F, Diot P, Gauthier F, et al. Influence of DNA on the Activities and Inhibition of Neutrophil Serine Proteases in Cystic Fibrosis Sputum. *Am. J. Respir. Cell Mol. Biol.* 2012;47:80–86.
9. Papayannopoulos V, Staab D, Zychlinsky A. Neutrophil Elastase Enhances Sputum Solubilization in Cystic Fibrosis Patients Receiving DNase Therapy. *PLoS ONE*. 2011;6:e28526.
10. Yang C, Montgomery M. Dornase alfa for cystic fibrosis. *Cochrane Database Syst. Rev.* [Internet]. 2018 [cited 2020 Mar 23];Available from: http://doi.wiley.com/10.1002/14651858.CD001127.pub4
11. Simmons JD, Freno DR, Muscat CA, Obiako B, Lee YL, Pastukh VM, et al. Mitochondrial DNA damage associated molecular patterns in ventilator-associated pneumonia: Prevention and reversal by intratracheal DNase I. *J. Trauma Acute Care Surg.* 2017;82:120–125.
12. Zitter JN, Maldjian P, Brimacombe M, Fennelly KP. Inhaled Dornase alfa (Pulmozyme) as a noninvasive treatment of atelectasis in mechanically ventilated patients. *J. Crit. Care*. 2013;28:218.e1-218.e7.
13. Cao B, Wang Y, Wen D, Liu W, Wang J, Fan G, et al. A Trial of Lopinavir–Ritonavir in Adults Hospitalized with Severe Covid-19. *N. Engl. J. Med.* 2020;NEJMoa2001282.
14. Liu F-C, Chuang Y-H, Tsai Y-F, Yu H-P. Role of Neutrophil Extracellular Traps Following Injury: *Shock*. 2014;41:491–498.
15. McIlroy DJ, Jarnicki AG, Au GG, Lott N, Smith DW, Hansbro PM, et al. Mitochondrial DNA neutrophil extracellular traps are formed after trauma and subsequent surgery. *J. Crit. Care*. 2014;29:1133.e1-1133.e5.
